# Effects of Salt Treatment Time on the Metabolites, Microbial Composition, and Quality Characteristics of the Soy Sauce Moromi Extract

**DOI:** 10.3390/foods11010063

**Published:** 2021-12-28

**Authors:** Sun Lee, Dong-Shin Kim, Yejin Son, Huong-Giang Le, Seung Wha Jo, Jungmi Lee, Yeji Song, Hyun-Jin Kim

**Affiliations:** 1 Department of Food Science & Technology, Gyeongsang National University, Jinju 52828, Korea; pedric98@naver.com; 2 Institute of Animal Medicine, Gyeongsang National University, Jinju 52828, Korea; feel567@naver.com (D.-S.K.); yejin7739@naver.com (Y.S.); 3 Division of Applied Life Sciences (BK21 Four), Institute of Agriculture and Life Science, Gyeongsang National University, Jinju 52828, Korea; giangcnshe@gmail.com; 4 Microbial Institute for Fermentation Industry, Sunchang 56048, Korea; tmdghk606@hanmail.net; 5 Sunchangjangryu Corp., Sunchang 56048, Korea; lovehus@hanmail.net (J.L.); syj4950@naver.com (Y.S.)

**Keywords:** metabolomics, microbial population, salt treatment time, sensory quality, soy sauce moromi extract

## Abstract

Salt is one of the most important factors for fermented foods, but the effect of salt treatment time on the quality of fermented foods has rarely been studied. In this study, the effect of different salt treatment times (0, 48, and 96 h) after the start of fermentation on the quality of the soy sauce moromi extract (SSME) was investigated. As the salt treatment time was delayed, the population of *Aspergillus oryzae*, *Lactobacillaceae*, and *Enterococcaecea* in SSME increased, whereas the population of *Staphylococcaceae* and *Bacillaceae* decreased, leading to changes in the enzymatic activity and metabolite profiles. In particular, the contents of amino acids, peptides, volatile compounds, acidic compounds, sugars, and secondary metabolites were significantly affected by the salt treatment time, resulting in changes in the sensory quality and appearance of SSME. The correlation data showed that metabolites, bacterial population, and sensory parameters had strong positive or negative correlations with each other. Moreover, based on metabolomics analysis, the salt treatment-time-related SSME metabolomic pathway was proposed. Although further studies are needed to elucidate the salt treatment mechanism in fermented foods, our data can be useful to better understand the effect of salt treatment time on the quality of fermented foods.

## 1. Introduction

Fermented foods produced via controlled microbial growth are important in the human diet because they are rich in nutrients and can be preserved for a long time [1]. Moreover, large cohort investigations and long-term prospective studies have revealed strong associations between the consumption of fermented foods and a reduction in the risk of obesity-related diseases, cardiovascular diseases, diabetes, and overall mortality [2,3]. Accumulating evidence has revealed that various living microorganisms and small metabolites of fermented foods contribute positively to these health benefits [4]. In addition to these health benefits, small metabolites produced by the fermentation of large molecules, such as proteins, carbohydrates, and lipids, from many food materials, such as meat, fish, soybeans, dairy, vegetables, cereals, and fruits, are associated with a diversity of flavors and unique tastes [5,6]. However, the metabolite profiles dramatically change depending on various fermentation factors, including microbial population, food materials, additional ingredients, and fermentation conditions, such as time and temperature [7].

Salt is one of the most important factors because it prevents harmful bacterial growth, contributes to sensory taste, and slows down the fermentation process to allow the flavor to develop further [8,9]. However, in recent years, many salt-fermented foods, such as soy sauce, doenjang, and miso, have been commercially produced by starter-added fermentation under controlled fermentation environmental conditions, including salt concentration and type, to quickly mass-produce fermented foods of the same quality [10]. Most studies on the effect of salt on the quality of fermented foods have been related to salt concentration [9,11]. In particular, metabolomics, which aids in understanding the dynamics of microbial communities and monitoring the changes in metabolites associated with the sensory and nutritional quality of fermented foods during the fermentation process [12], has been applied to investigate the effect of salt concentration on the quality of fermented foods, including kimchi [13] and soy sauce [14].

Among salt-fermented foods, soy sauce is traditionally produced using moromi prepared with koji at 15–30 °C and 18–22% salt content, but commercial soy sauce is manufactured at higher temperature and lower salt content than the traditional method to shorten production time [10]. Many soy sauce moromi fermentation studies have been conducted to find the optimal fermentation conditions for commercial soy sauce production [10,15], but the effect of salt treatment time on the quality of soy sauce moromi has been rarely studied.

Therefore, in this study, to better understand the effect of different salt treatment times on the quality of the soy sauce moromi extracts (SSMEs), metabolite profiles of SSMEs with different salt treatment times (0, 48, or 96 h) were analyzed using ultra-high-performance liquid chromatography (UPLC)-quadrupole (Q)-time-of-flight (TOF) mass spectrometry (MS) (UPLC-Q-TOF MS) and gas chromatography (GC)/MS. In addition, their general characteristics, sensory qualities, microbial populations, and metabolite profiles were investigated, and the correlation with the metabolite profiles was evaluated.

## 2. Materials and Methods

### 2.1. Preparation of SSME and Salt Treatment

Soy sauce moromi was prepared according to the method described by Hoang et al. with modification [10]. Roasted soybean powder was mixed with water and soy koji prepared with *Aspergillus oryzae* SRCM102487 (0.3%, *w*/*w*). The mixture with a final soluble solid content of 23 °C Brix was fermented at 45 °C for 5 d. During fermentation, salt was added to the fermentation mixture to a final concentration of 10% at different times (0, 48, or 96 h). After fermentation, the fermented moromi was filtered using Whatman No. 2 filter paper and the filtered liquid was used as SSME in this study.

### 2.2. Determination of General Characteristics

To evaluate the general characteristics of SSMEs, pH, salt content, amino-type nitrogen (AN), color, total acidity, and reducing sugar contents were measured. The salt content of the SSMEs was determined using a salinometer (Atago Co. LTD., Tokyo, Japan). The AN content was measured using the formol titration method [16]. The sample mixed with distilled water and formaldehyde solution (pH 8.3) was titrated with sodium hydroxide (NaOH) (0.1 N), and the amount of NaOH was used to calculate the AN content. For total acidity, the sample (1 mL) was mixed with distilled water (9 mL) followed by titration with 0.01 N NaOH at pH 8.3 and expressed as % of lactic acid [12]. The reducing sugar content was determined using a modified 3, 5-dinitrosalicylic acid (DNS) method [17] with minor modifications. SSME (400 μL) mixed with 1.2 mL DNS solution was heated at 90 °C for 5 min, and then the absorbance was measured at 540 nm. Glucose was used as the standard.

### 2.3. Determination of Protease and Amylase Activities

The protein content of SSMEs was determined using the Bradford method [18], and albumin was used as the standard. Protease activity was determined using the Anson method [19] with minor modifications. SSME (40 µL) mixed with 400 mL of 0.6% casein solution was incubated at 37 °C for 10 min and stopped by adding 0.44 M trichloroacetic acid solution (800 μL). After centrifugation, the supernatant (400 μL) was mixed with 1 mL of 0.55 M sodium carbonate and 200 µL of Folin reagent. The absorbance of the reaction solution was determined at 660 nm, and tyrosine was used as the standard. One unit (U) was defined as the amount of enzyme that produced 1 μg of tyrosine in 1 min at 37 °C, and protease activity was expressed as U/mg protein. The α-amylase activity was assayed by the DNS procedure [20], using 3% soluble starch as a substrate. Soluble starch (100 µL) was incubated for 30 min at 30 °C with 100 µL of diluted SSME. The reaction was stopped by adding DNS (600 µL) and heated in boiling water for 10 min. After cooling on ice, the absorbance was measured at 540 nm, and glucose was used as the standard. One U of α-amylase activity was defined as the amount of α-amylase that induced a change in 1 mg of glucose in soluble starch solution for 1 min at 30 °C, and α-amylase activity was expressed as U/mg protein.

### 2.4. Microbial Analysis

Total fungal and bacterial counts of SSMEs were analyzed using the culture-dependent method and microbial populations were analyzed using 16s rRNA sequencing [21]. For the total viable bacterial count, 1 mL of each SSME was homogenized with 9 mL of sterilized peptone water in a test tube. The homogenized samples were spread onto a plate count agar after dilution using sterilized peptone water and then incubated at 37 °C for 24 h to determine the total viable bacterial numbers. The total number of viable bacteria was expressed as log colony forming unit (CFU)/mL sample. For total fungal count, 1 mL of each SSEM was spread onto 3M Petrifilm yeast and mold count plate (3M Food Safety, St. Paul, MN, USA) and incubated at 30 °C for 2 days. The total viable count of fungi is expressed as CFU/mL.

The DNA extracted from SSMEs using the DNeasy PowerSoil kit (Qiagen, Hilden, Germany) was amplified using 16S universal primers targeting V3 and V4 regions in the FastStart High Fidelity polymerase chain reaction (PCR) System (Roche, Basel, Switzerland) [21]. DNA library was constructed using Genome Sequencer (GS) FLX plus (454 Life Science, Brandford, CT, USA) according to the manufacturer’s instructions. The library was quantified using the Illumina MiSeq standard Kit v.3 (Illumina, San Diego, CA, USA). The 16S amplicon sequences of each sample were assessed using Mothur v.1.36. software using the MiSeq according to the manufacturer’s instructions. The sequences were aligned, clustered, and identified using the Silva reference alignment v.12350. The taxonomy and bacteria counts in each sample were determined [22]. The relative bacteria number of each sample was calculated in the taxonomic assignments at the family, order, genus, and species levels. The α-diversity with the Chao1 and Shannon indices was determined using the Mothur program.

### 2.5. Sensory Evaluation

Quantitative descriptive analysis was performed by 8 trained panelists aged 20 to 31 years. All panelists had previous experience with sensory evaluation for more than 2 weeks. Before sensory evaluation, the panelists discussed a series of taste reference solutions, including citric acid (0.2%) for sourness, sucrose (5%) for sweetness, sodium chloride (NaCl) (0.5%) for salty taste, quinine (0.0025%) for bitterness, monosodium glutamate (MSG) (0.3%) for umami taste, ethanol (20%) for ethanolic odor, acetic acid (3%) for acetic acid odor, and commercial soy sauce (Agricultural Sunchang Jangryu Corp., Sunchang, Korea) for soy sauce odor. The intensity of each sensory quality was rated on a 15 cm line scale labeled “very weak” and “very strong” with 0.5 cm anchors on the left and right sides [9].

### 2.6. GC/MS-Based Metabolomic Analysis

The non-volatile and volatile metabolites of SSMEs were analyzed using GC/MS following the method of Kim et al. [23]. For GC/MS analysis of non-volatile metabolites, lyophilized SSME (*n* = 5) was homogenized with 80% methanol using a bullet blender (Next Advance, Troy, NY, USA). The extracts were dried using a CentriVap concentrator SpeedVac (Labconco Co., Kansas City, MO, USA). For derivatization, the residues dissolved in 150 μL of methoxyamine hydrochloride containing pyridine and dicyclohexyl phthalate as an internal standard (IS) were incubated at 37 °C for 90 min and then derivatized by adding 150 μL N, O-bis(trimethylsilyl)trifluoroacetamide (BSTFA) at 70 °C for 30 min.

For volatile metabolite analysis, SSMEs (1 mL) with NaCl (1 g) and 2-methyl-1-pentanol as an IS were placed in a vial with a septum cap and heated at 55 °C for 10 min. After heating, volatile metabolites in the head space were absorbed using a solid-phase microextraction (SPME) fiber (50/30 μm divinylbenzene/carboxen/polydimethylsiloxane Stableflex; Sigma-Aldrich, Saint Louis, MO, USA) at 55 °C for 10 min. The derivatized non-volatile metabolites and the absorbed volatile metabolites were analyzed using a GC/MS system (Shimadzu Corp., Kyoto, Japan) equipped with a DB-5MS column (30 m × 0.25 mm, 0.25 μm film thickness, Agilent Technologies, Santa Clara, CA, USA) and a DB-Wax column (30 m × 0.25 mm, 0.25 μm film thickness, Agilent Technologies), respectively. For analysis of volatile metabolites, the injector temperature was set to 270 °C, and the oven temperature was programmed as follows: 50 °C (isothermal for 2 min), 50–180 °C for 3 min^−1^, 180–300 °C at 10 °C min^−1^, and 300 °C (isothermal for 3 min). For analysis of non-volatile metabolites, the injector temperature was set to 200 °C and the oven temperature was programmed as follows: 70 °C (isothermal for for 2 min), 70–320 °C at 10 °C min^−1^, and 320 °C (isothermal for 5 min). Helium was used as the carrier gas at a flow rate of 0.95 mL/min. The effluent was detected using a GCMS-TQ 8030 MS (Shimadzu Corp., Kyoto, Japan) with electron ionization at 70 eV, ion source temperature of 230 °C, and interface temperature of 280 °C. Data were monitored at *m/z* 45–550.

### 2.7. UPLC-Q-TOF MS-Based Metabolomic Analysis

SSME metabolite profiles were analyzed using UPLC-Q-TOF MS (Waters, Milford, MA, USA), following a previously described method with modifications [23]. To extract SSME metabolites, lyophilized SSME (*n* = 5) was homogenized with 80% methanol containing terfenadine as an IS using a bullet blender. After centrifugation, the supernatant was injected into an Acquity BEH C18 column (2.1 mm × 100 mm, 1.7 µm; Waters, Milford, MA, USA), equilibrated with water containing 0.1% formic acid (A), and set at a column temperature of 40 °C. The metabolites were eluted with a linear gradient of acetonitrile containing 0.1% formic acid (B) at a flow rate of 0.35 mL/min. The eluents were detected using Q-TOF MS with positive electrospray ionization (ESI). The optimal conditions for MS analysis were set at sampling cone voltage of 40 V, capillary voltage of 3 kV, desolvation temperature of 400 °C, source temperature of 120 °C, and desolvation gas flow rate of 800 L/h. Leucine-enkephalin ([M + H] = 556.2771) was used as the lock mass. MS data were obtained with a scan range of 50 to 1500 *m/z*. The MS/MS data were obtained using collision energy ramps from 10 to 40 eV.

### 2.8. Organic Acid Analysis

For organic acid analysis, lyophilized SSME (*n* = 5) was homogenized with distilled water containing with 5% aqueous meta-phosphoric acid by sonication. After centrifugation, the supernatants were analyzed by HPLC (Shimadzu Corp., Kyoto, Japan) equipped with a photodiode array detector (Shimadzu Corp., Kyoto, Japan). A Triart C18 column (250 mm × 4.6 mm, 4.6 mm I.D., 5 μm; YMC Co. Ltd., Kyoto, Japan.) was used for separation of organic acids. The column temperature was set at 40 °C. 0.1% aqueous phosphoric acid was used as a mobile phase. The flow rate was 1 mL/min and elutes were detected at 220 nm.

### 2.9. Data Processing

Data obtained using GC/MS were aligned with retention time window of 0.1–0.05 min and normalized with the IS. Metabolites were identified using GC/MS databases (NIST 11 and Wiley 9 mass spectral libraries), and retention indices were calculated using *n*-alkanes and standard chemicals. Metabolites analyzed by UPLC-Q-TOF MS were processed using MarkerLynx software (Waters, Milford, MA, USA) for collection, alignment, and normalization. The data were aligned with a 0.05 Da mass window and 0.2 min retention time window. All data were normalized to an IS. Metabolites were identified using the UNIFI software (Waters, Milford, MA, USA) connected to various online databases and the METLIN database (www.metlin.scripps.edu (accessed on 9 October 2021)).

### 2.10. Statistical Analysis

Processed data sets were analyzed with multivariate statistics using SIMCA-P+ v.14.0.1 (Umetrics, Umea, Sweden) and the comparison of data between SSMEs was visualized using partial least squares discriminant analysis (PLS-DA). Normalized chromatogram intensities of metabolites and all data, including pH, acid value, color, reducing sugars, salt content, enzymatic activity, total bacterial count, and sensory evaluation were statistically analyzed using one-way analysis of variance (ANOVA) with Duncan’s test (*p* < 0.05) using SPSS v.23.0 (SPSS Inc., Chicago, IL, USA). Pearson’s correlation coefficients between variables, including the percentage of microbial population, sensory qualities, and relative abundances of identified metabolites were calculated and visualized using the R software.

## 3. Results and Discussion

### 3.1. Microbial Analysis

**Figure 1:**
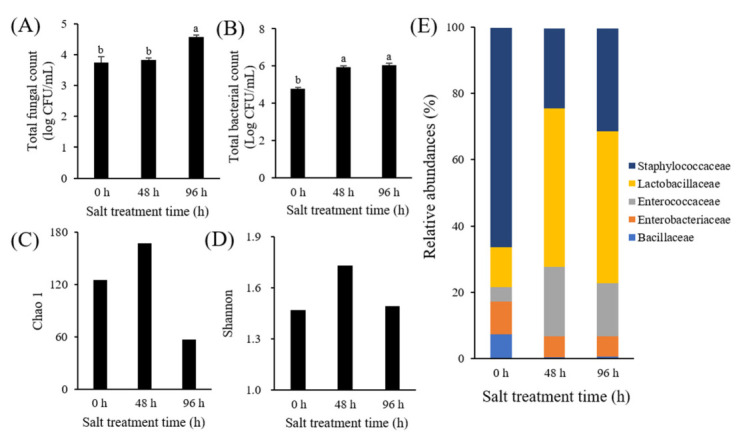
Microbial analysis of soy sauce moromi extracts (SSMEs) at different salt treatment time: Total fungal count (**A**), total bacteria count (**B**), Chao1 total richness (**C**), Shannon diversity index (**D**), and bacteria communities at family levels (**E**). Total fungal and bacterial counts of SSMEs were analyzed using culture-dependent method and microbial populations were analyzed using 16s rRNA sequencing. Different letters in each bar means significant differences by Duncan’s test (*p* < 0.05).

The total fungal count of SSME increased from 3.75 CFU/mL (0 h) to 4.57 CFU/mL (96 h) with a delay in the salt treatment time (Figure 1A), while the total bacterial count increased from 4.7 log CFU/mL (0 h) to 6.1 log CFU/mL (96 h) (Figure 1B). However, there was no significant difference in the total fungal and bacterial counts between the 48 and 96 h SSMEs. This result indicated that the growth of some bacteria and fungi was suppressed by treatment with 10% salt. In particular, although *A. oryzae* inoculated for producing SSMEs is known to be a halophilic fungus, its growth was also suppressed by the 10% salt treatment because high salt treatment inhibits the development and conidia formation of *A. oryzae* via the corresponding intracellular accumulation of arginine and oleic acid [24]. To analyze the effect of salt on the bacterial community of SSMEs, the SSME microbiota were analyzed using 16S rRNA sequencing (Figure 1C–E). The Chao1 and Shannon indices for evaluating the richness and diversity of the microbiota showed that the richness and diversity of the microbial community increased at 48 h SSME compared to that at 0 h SSME. However, both increased indices decreased at 96 h SSME (Figure 1C,D). The analysis of the bacterial community at the family level revealed that five families, including *Staphylococcaceae*, *Lactobacillaceae*, *Enterococcaecea*, *Enterobacteriaceca*, and *Bacillaceae*, were considered as major components of the SSME microbiome. The populations of *Staphylococcaceae*, *Enterobacteriaceae*, and *Bacillaceae* decreased from 66, 9.8, and 7.4% at 0 h SSME to 24.2, 6.3, and 0.4% at 48 h SSME, respectively, whereas the populations of *Lactobacillaceae* and *Enterococcaecea* increased from 12.1 and 4.4% at 0 h SSME to 47.6 and 21.0% at 48 hSSME, respectively. However, no further changes were observed in the 96 h SSME. This result indicated that the growth of non-halophilic bacteria, such as *Lactobacillaceae* and *Enterococcaecea*, was suppressed by 10% salt treatment, whereas the growth of halophilic bacteria, such as *Staphylococcaceae* and *Bacillaceae*, was inhibited by low salt concentrations. Similar results were observed in pickles [25] and meju [26]. These bacterial families are frequently detected in various fermented soy foods, such as doenjang and soy sauce [27,28]. Among them, *Staphylococcaceae*, which has a strong survival ability in a hypertonic environment, including high salt concentrations [27], are considered food-borne bacterial pathogens, but most *Staphylococcaceae* species, except *S. aureus*, are harmless to humans [27]. A previous study reported that *Staphylococcus* species produce volatile fatty acids, which contribute to the flavor of fish sauce in salt-fermented seafood [29]. *Lactobacillaceae* and *Enterococcaceae*, a large group of non-halophilic lactic acid bacteria, are related to antimicrobial antagonism as well as cheese taste and flavor [30]. In particular, some *Enterococcaceae* members, including *E. faecalis* and *E. faecium*, produce bacteriocins, which are antibacterial peptides that exhibit inhibitory activity against food spoilage and growth of food-borne pathogens, including *Bacillus* ssp., *Listeria* spp., and *Clostridium* ssp. [31]. Therefore, the increased population of both bacteria suppressed the growth of halophilic bacteria, such as *Staphylococcaceae* and *Bacillaceae*, but *Enterobacteriaceae* was not seriously affected.

### 3.2. General Characteristics

To evaluate the general characteristics of SSMEs, their pH, total acidity, AN content, salt content, reducing sugar content, and color were measured (Table 1). As the salt treatment time was delayed, the pH of the SSME decreased slightly from 5.10 (0 h) to 4.96 (49 and 96 h), whereas the acid value increased from 1.56 (0 h) to 2.32 (49 h) and 2.65 (96 h). Moreover, the AN content of 48 and 96 h SSME (781.98 mg%) was two times higher than that of 0 h SSME (389.84 mg %), whereas the reducing sugar content decreased with delay in salt treatment time, and the amount of 0 h SSME (2.81%) was 5.7 and 8.3 times greater than that at 48 h (0.49%) and 96 h (0.34), respectively. The color of SSME was also altered by different salt treatment times. L* and a* values increased slightly with a delay in salt treatment time, but the b* value of 48 and 96 h SSME was more than two times higher than that of 0 h SSME. The brown color produced by the Maillard reaction between reducing sugars and amino acids produced during fermentation was inhibited by the addition of NaCl because an increase in NaCl concentration might change the ionization state in the Maillard reaction, resulting in the inhibition of melanoidin formation [32]. Similar results were observed for doenjang with different salt concentrations [33] and the Maillard reaction model system [34].

### 3.3. Enzymatic Activity

Protease and α-amylase activities often provide indirect information on the quality of fermented foods, including soy sauce, because of its close relationship with the production of a unique and desirable flavor in fermented foods [35]. Protease activity increased with a delay of the salt treatment time, and the activity of 96 h SSME (2.51 U/mg protein) was two times higher than that of 0 h SSME (1.27 U/mg protein). However, amylase activity decreased with a delay of the salt treatment time, and the activities of 48 and 96 h SSMEs (0.52 and 0.45 U/mg protein, respectively) were about 4 times lower than that of 0 h SSME (1.99 U/mg protein) (Table 2). Enzymatic activities of SSMEs, such as deonjang [36] and fermented pickles [25] are mainly affected by *A. oryzae* and various other bacteria. In particular, protease activity can be positively affected by *A. oryzae* SRCM102487, which is used for moromi production and has a relatively high protease activity, whereas a decrease in amylase activity with a delay in salt treatment time was mainly affected by a decrease in *Staphylococcaceae* and *Bacillaceae* species, which have relatively high amylase activities compared to *Lactobacillaceae* and *Enterococcaecea* [37].

### 3.4. Sensory Evaluation

**Figure 2:**
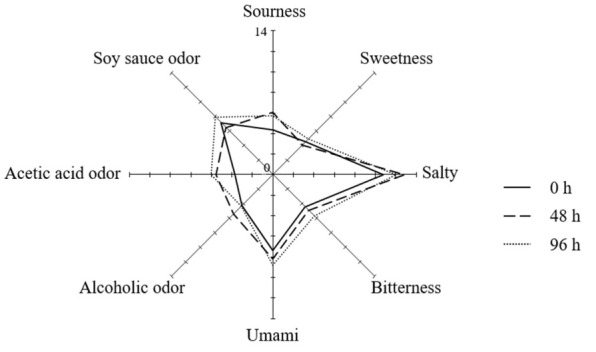
Sensory qualities of SSMEs at different salt treatment time. The intensity of each sensory quality was rated on a 15 cm line scale, labeled “very weak” and “very strong” with 0.5 cm anchors on the left and right sides.

To evaluate the sensory characteristics of SSMEs according to the salt treatment time, quantitative descriptive analyses for five tastes (sour, sweet, salty, bitter, and umami) and three flavors (alcoholic odor, acetic acid odor, and soy sauce odor) were performed (Figure 2). As the salt treatment time was delayed, the sensory intensities of most of the taste parameters increased. In particular, the sourness, bitterness, umami taste, and acetic acid odor increased from 4.4, 4.4, 7.4, and 3.7 at 0 h SSME to 5.7, 5.7, 8.8, and 6.1 at 96 h SSME, respectively. Many studies have reported that the sensory characteristics of fermented foods are significantly affected by the small metabolites produced by microorganisms [38,39]. To confirm this, the metabolite profiles of SSMEs were analyzed, and their correlations with the sensory qualities were evaluated.

### 3.5. Metabolome Analysis

**Figure 3:**
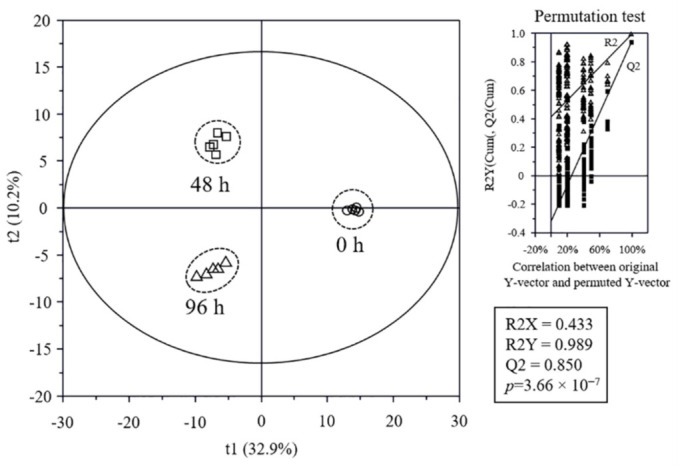
Partial least-squares discriminant analysis (PLS-DA) score plot of soy sauce moromi metabolites analysis and quality parameters. Metabolites were analyzed using gas chromatography (GC)-mass spectrometry (MS), ultra-high-performance liquid chromatography (UPLC)-quadrupole (Q)-time-of-flight (TOF) (UPLC-Q-TOF) MS, and high-performance liquid chromatography (HPLC). The qualification of the PLS-DA model was evaluated using R2X, R2Y, Q2, and *p*-value and validated using cross validation with a permutation test.

**Figure 4:**
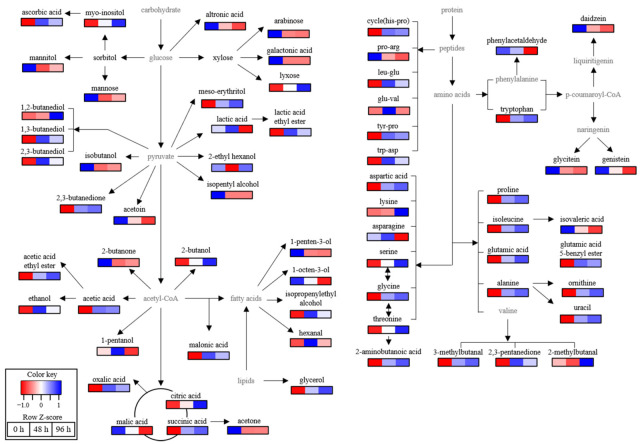
Proposed SSME-metabolomic pathway related to salt treatment time. The heat maps drawn by R software with ggplot2 showed the relative abundance of identified metabolites. The heat map color represented the z-score transformed raw data of metabolites with the significant difference among groups. Blue and red indicate the increase and decrease in metabolite levels, respectively.

The non-volatile and volatile metabolite profiles in SSMEs with different salt treatments were analyzed using GC-MS, UPLC-QTOF-MS, or HPLC (Figure S1) and statistically compared. The discrimination of SSMEs was visualized by PLS-DA score plot (Figure 3) and loading scatter plot (Figure S2). The goodness of fit (R2X = 0.433; R2Y = 0.989), predictability (Q2 = 0.850), *p*-values (3.66 × 10^−7^), and the cross-validation determined by the permutation test of the PLS-DA model was statistically acceptable (Figure 3). In the score plot, the 0 h SSME was clearly separated from other SSMEs by t(1), and 48 and 96 h SSMEs were also clearly separated from each other by t(2). The VIP and *p*-values of all metabolites were analyzed to find the metabolites contributing to the differences among SSMEs (Table 3, Tables S1 and S2). Among them, 68 metabolites, including 4 aldehydes, 13 alcohols, 6 ketones, 2 esters, 13 amino acids, 6 peptides, 9 sugars, 10 acidic compounds, 3 isoflavones, and 2 other compounds, with higher VIP (VIP ≥ 0.83) and lower *p*-values (≤0.05) were identified as the major metabolites contributing to the differences in the groups on the PLS-DA score plot. The normalized chromatogram intensities were compared according to the different salt treatment times (Figure 4), and their fold changes were calculated against 0 h SSME (Table 3). Metabolite analysis data showed significant differences in the small metabolite profiles between SSMEs, but there was no significant difference in the fold changes of most metabolites between 48 h and 96 h SSMEs.

Among non-volatile compounds, the amounts of most amino acids and peptides produced by the proteases of microorganisms during fermentation increased with a delay in salt treatment time, whereas aspargine, pro-arg, and cyclo(his-pro) decreased. The contents of ornithine, proline, aspartic acid, lysine, 2-aminobutyric acid, isoleucine, glutamic acid, alanine, tyr-pro, and leu-glu in 96 h SSME were 2–3.4 times higher than those of 0 h SSME, while the contents of glutamic acid 5-benzyl ester and trp-asp in 96 h SSME were 19 and 5.7 times higher, respectively. The increase in these amino acids and peptides contributed to the sensory quality of SSMEs because these amino acids and peptides are generally involved in the production of brown color, volatile compounds, and taste-related compounds [40,41] via the Maillard reaction [41] or the Ehrlich reaction [40]. In particular, increased glutamic acid-containing compounds contributed to an increase in the umami taste of SSMEs [41], while accumulated brown-colored compounds led to an increase in the bitterness of SSMEs [42].

In addition to amino acids and peptides, the profiles of volatile compounds, such as alcohols, aldehydes, ketones, esters, and some acidic compounds, changed significantly with increasing salt treatment time. Although there was no rich aroma similar to soy sauce due to the short fermentation period in this study, volatile compounds known as key aroma compounds of soy sauce, including 2-methylbutanal, 3-methylbutanal, phenylacetaldehyde, acetic acid, and ester compounds, were detected, and their contents increased with a delay in the salt treatment time. In particular, the content of acetic acid, which is produced by lactic acid bacteria during fermentation [43], was 6.7 times higher in 96 h SSME than 0 h SSME, contributing to the increase in the sourness and sour odor of SSMEs. Moreover, acetic, lactic, and glutamic acids can react with alcohols to produce esters, such as acetic acid ethyl ester, lactic acid ethyl ester, and glutamic acid 5-benzyl ester, which are abundant aroma producing compounds in various sauces [40]. In 96 h SSME, their amounts were 5.3, 2.7, and 19 times higher, respectively, than those of 0 h SSME, contributing to a positive deep aroma to the SSME. However, acetic acid-related ketones, such as acetoin and acetone, decreased with a delay in the salt treatment time, while 2,3-buandedione and 2,3-pentanedione increased. These ketones are responsible for the pleasing aroma of soy sauce and koji products [44], but they are intermediate unstable compounds and are reduced to alcohols [45]. In addition to aldehydes and ketones, alcohols are generally produced via lipid oxidation, as well as the Maillard reaction and the Ehrlich reaction during fermentation. In particular, butanediols, which clearly negatively affect the sensory quality of soy sauces [46], increased with a delay in the salt treatment time, and the contents of 1,2-butanediol and 1,3-butanediol in 96 h SSME were 67.3 and 5.4 times higher, respectively, than those of 0 h SSME, while the 1,2-butanol level of 96 h SSME was 6.1 times higher than that of 0 h SSME.

Unlike amino acids, peptides, and volatile compounds, most sugars and soybean secondary metabolites with various health benefits [1,5] were minor compounds of SSMEs, and their contents were reduced by up to six times with a delay in the salt treatment time. A similar result was reported for doenjangs with different salt concentrations [47].

### 3.6. Correlation between Metabolites, Bacterial Populations, and Sensory Parameters

**Figure 5:**
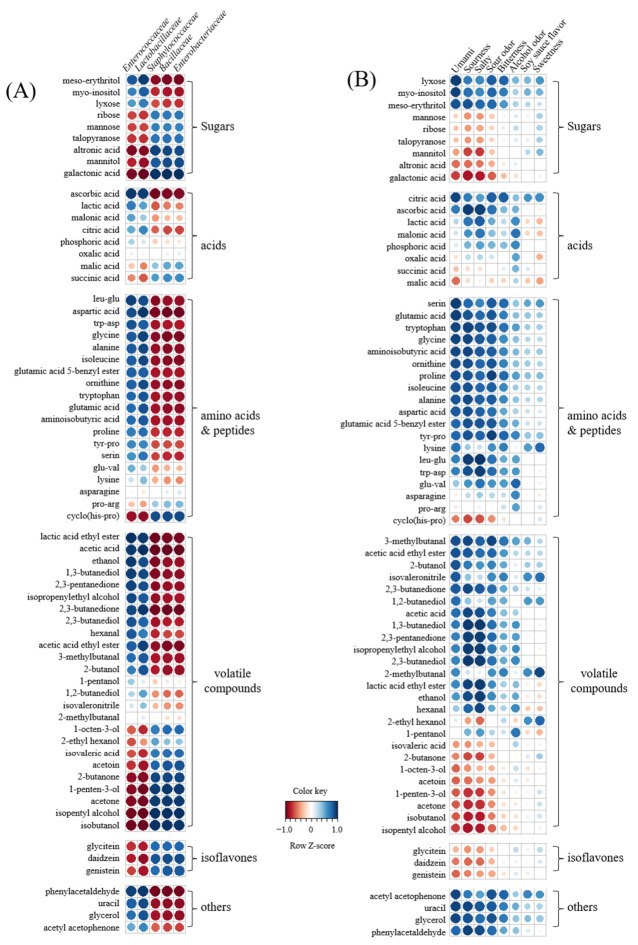
Correlation between microorganisms and metabolites (**A**) and sensory quality and metabolites (**B**). Pearson correlation coefficient was calculated and visualized using the R software. Correlation heat map colors represent the correlation coefficients, and blue and red colors on a red–blue color scale indicate the positive and negative correlations, respectively.

Correlations between metabolites, bacterial populations, and sensory parameters were analyzed using the Pearson correlation coefficient and visualized using heatmaps (Figure 5). In the correlation between metabolites and bacterial populations (Figure 5A, Tables S3 and S4), *Enterococcaceae* and *Lactobacillaceae* were increased with a delay in the salt treatment time and had a positive correlation (r > 0.50) with increased metabolites in 48 and 96 h SSMEs compared to 0 h SSME but had a negative correlation (r < −0.45) with decreased metabolites, except arabitol, malonic acid, lactic acid, oxalic acid, glu-val, lysine, pro-arg, 1-pentanol, 1,2-butanediol, 5-methyl-3-hexen-2-one, and 2-methylbutanal. On the other hand, *Staphylococccaceae*, *Bacillaceae*, and *Enterobacteriaceae* decreased with a delay in the salt treatment time and had exactly opposite correlations with metabolites that were positively or negatively correlated with *Enterococcaceae* and *Lactobacillaceae*. Moreover, the correlation data between metabolites and sensory parameters showed that umami taste, sourness, salty taste, acetic odor, and bitterness had strong positive or negative correlations similar to those of metabolites that had strong correlations with *Enterococcaceae* and *Lactobacillaceae* (Figure 5B). However, alcohol odor, soy sauce odor, and sweetness had no or weak correlation with most metabolites, except for a few metabolites. Soy sauce odor and sweetness were positively correlated with 5-methyl-3-hexen-2-one, 1,2-butanediol, 2-methylbutanal, 2-ethyl hexanol, and sugar alcohols, except mannitol (r ≥ 0.47), while alcoholic odor was positively correlated with lactic acid, malonic acid, glu-val, lysine, tyr-pro, 1,3-butandiol, isopheylethyl alcohol, 2,3-buanediol, hexanal, and 1-pentanol (r ≥ 0.60). As the salt treatment time was delayed, the population of bacteria with less salt sensitivity increased, which could enrich the taste and flavor of fermented foods by increasing various metabolites related to sensory quality. Therefore, these results indicate that salt treatment time is an important factor contributing to the sensory quality of fermented foods, including other fermentation factors, such as salt concentration and fermentation period [7,10,11].

## 4. Conclusions

In this study, the effect of different salt treatment times (0, 48, and 96 h) after the start of fermentation on the quality of SSMEs was investigated using metabolomics, microbial analysis, and general characteristics. As the salt treatment time was delayed, the populations of *A. oryzae* and non-halophilic bacteria, including *Lactobacillaceae* and *Enterococcaecea*, in SSME increased, whereas the population of halophilic bacteria, such as *Staphylococcaceae* and *Bacillaceae*, decreased, leading to changes in the protease and amylase activities and metabolite profiles. In addition, salt treatment moderated the Maillard reaction. In particular, the amino acid, peptide, volatile compound, acidic compound, sugar, and secondary metabolite contents were significantly affected by the salt treatment time. Among these metabolites, umami taste-related compounds and aromatic compounds, including esters, acetic acid, aldehydes, and ketones, accumulated significantly with a delay in the salt treatment time, resulting in an increase in the umami taste, bitter taste, sourness, acetic acid odor, alcoholic odor, and brown color of SSME. The correlation data showed that metabolites, bacterial population, and sensory parameters had strong positive or negative correlations with each other. Moreover, based on the metabolomic analysis, the SSME metabolomics pathway related to salt treatment time was proposed. Although further studies on the optimization of salt treatment time and the mechanism of salt effect on chemical reactions in SSME are needed, the data presented in this study can be useful to better understand the effect of salt treatment time on the quality of fermented foods.

## Figures and Tables

**Table 1:** General characteristics of soy sauce moromi extracts with different salt treatment time.

		Salt Treatment Time (h)		
		0	48	96
pH value		5.12 ± 0.04 ^a^	4.71 ± 0.02 ^b^	4.72 ± 0.03 ^b^
Acid value (%)		1.56 ± 0.010 ^c^	2.32 ± 0.05 ^b^	2.65 ± 0.20 ^a^
Amino-type nitrogen (mg %)		389.84 ± 15.98 ^b^	781.98 ± 73.24 ^a^	735.85 ± 55.94 ^a^
Salt content (%)		6.83 ± 0.42 ^b^	7.48 ± 0.17 ^a^	7.40 ± 0.14 ^a^
Reducing sugar (%)		2.81 ± 0.19 ^a^	0.49 ± 0.08 ^b^	0.34 ± 0.04 ^b^
Color	L*	24.70 ± 0.34 ^b^	26.73 ± 0.07 ^a^	26.74 ± 0.05 ^a^
	a*	1.05 ± 0.15 ^b^	1.64 ± 0.03 ^a^	1.67 ± 0.03 ^a^
	b*	2.19 ± 0.18 ^c^	4.96 ± 0.06 ^b^	5.15 ± 0.04 ^a^
	Brown index	12.17 ± 1.37 ^c^	24.63 ± 0.30 ^b^	25.56 ± 0.18 ^a^

L*, lightness; a*, redness; b*, yellowness; Browning index (BI) of samples was calculated by following equation: BI = [100(*x* – 0.31)]/0.17, where *x* = (a* + 1.75*L*)/(5.645L* + a* – 3.012b*); Different letters in each row means significant differences by Duncan’s test (*p* < 0.05).

**Table 2:** General characteristics of soy sauce moromi extracts with different salt treatment time.

Enzymatic Activity (U/mg Protein)	Salt Treatment Time (h)		
	0	48	96
Amylase activity	1.99 ± 0.02 ^a^	0.52 ± 0.02 ^b^	0.45 ± 0.00 ^c^
Protease activity	1.27 ± 0.11 ^b^	1.70 ± 0.03 ^b^	2.51 ± 0.54 ^a^

Different letters in each row means significant differences by Duncan’s test (*p* < 0.05).

**Table 3:** Identification of major metabolites contributing to the separation of samples on the PLS-DA score plots and their fold change.

	Compounds	VIP	*p*-Value	Fold Change (*vs*. 0 h)	
				48 h	96 h
sugars	meso−erythritol	1.25	2.27 × 10^−8^	2.32	2.69
	lyxose	1.26	9.31 × 10^−4^	1.62	2.25
	myo−inositol	1.39	2.62 × 10^−6^	1.46	1.86
	mannitol	1.23	7.77 × 10^−6^	−1.97	−1.59
	altronic acid	1.22	9.74 × 10^−6^	−2.06	−3.46
	mannose	0.89	0.02	−	−4.90
	arabinose	0.88	0.01	−5.06	−5.97
	talopyranose	0.92	9.15 × 10^−3^	−7.96	−6.09
	galactonic acid	1.23	1.58 × 10^−11^	−	−
acidic compounds	acetic acid	1.24	3.93 × 10^−12^	7.09	6.74
	ascorbic acid	1.29	1.66 × 10^−7^	6.86	5.34
	malonic acid	1.86	2.33 × 10^−4^	2.18	1.87
	citric acid	1.32	1.23 × 10^−3^	1.18	1.43
	succinic acid	1.14	9.15 × 10^−3^	1.54	1.04
	oxalic acid	1.66	6.62 × 10^−4^	1.38	−1.08
	phosphoric acid	1.43	0.04	1.62	−1.34
	lactic acid	1.83	1.92 × 10^−5^	1.17	−1.41
	isovaleric acid	1.13	1.15 × 10^−3^	−1.37	−1.72
	malic acid	1.83	2.56 × 10^−6^	−1.36	−2.26
amino acids and peptides	glutamic acid 5-benzyl ester	1.05	9.37 × 10^−4^	21.65	19.00
	trp−asp	1.23	2.72 × 10^−4^	8.40	5.74
	ornithine	1.16	1.75 × 10^−5^	2.94	3.41
	proline	0.96	4.51 × 10^−3^	2.71	3.00
	aspartic acid	1.24	1.04 × 10^−9^	2.61	2.92
	2−aminobutyric acid	1.11	1.98 × 10^−4^	2.29	2.61
	lysine	1.67	4.50 × 10^−4^	1.10	2.59
	isoleucine	1.18	5.68 × 10^−6^	2.26	2.57
	glutamic acid	1.22	9.45 × 10^−6^	2.12	2.53
	alanine	1.15	1.84 × 10^−5^	2.29	2.50
	tyr-pro	0.83	3.23 × 10^−2^	2.64	2.46
	leu-glu	1.30	8.52 × 10^−6^	3.00	2.33
	glycine	1.19	1.22 × 10^−6^	1.60	1.72
	serin	1.31	2.17 × 10^−4^	1.25	1.48
	tryptophan	1.10	1.68 × 10^−4^	1.37	1.44
	glu-val	1.32	3.69 × 10^−2^	2.57	1.05
	pro-arg	1.13	4.88 × 10^−2^	−1.21	−3.16
	asparagine	1.62	8.61 × 10^−3^	1.34	−4.29
	cyclo(his−pro)	1.11	6.26 × 10^−5^	−	−
alcohols	1,2-butanediol	1.92	1.76 × 10^−12^	8.36	67.38
	2-butanol	1.49	4.76 × 10^−3^	3.69	6.16
	1,3-butanediol	1.31	2.09 × 10^−6^	7.58	5.46
	glycerol	1.21	2.14 × 10^−5^	1.71	2.02
	isopropenylethyl alcohol	1.35	2.06 × 10^−5^	2.15	1.67
	ethanol	1.48	2.46 × 10^−4^	2.09	1.59
	2,3-butanediol	1.34	1.21 × 10^−4^	1.61	1.33
	2-ethyl hexanol	2.02	2.32 × 10^−7^	−2.03	1.05
	1-pentanol	1.93	2.23 × 10^−4^	1.19	−1.14
	1-octen-3-ol	1.46	5.54 × 10^−6^	−1.20	−1.57
	isobutanol	1.24	1.07 × 10^−9^	−3.25	−2.62
	1-penten-3-ol	1.19	1.06 × 10^−7^	−3.28	−3.49
	isopentyl alcohol	1.23	3.22 × 10^−13^	−5.01	−4.93
ketones	2,3-butanedione	1.21	7.17 × 10^−9^	5.07	5.49
	2,3-pentanedione	1.29	1.08 × 10^−5^	4.58	3.40
	acetyl acetophenone	1.36	6.85 × 10^−4^	1.11	1.27
	acetoin	1.27	1.32 × 10^−5^	−1.36	−1.69
	2-butanone	1.15	1.75 × 10^−5^	−2.21	−1.99
	acetone	1.18	3.79 × 10^−7^	−3.20	−3.12
aldehydes	3-methylbutanal	1.08	3.05 × 10^−4^	1.65	1.77
	hexanal	1.77	2.61 × 10^−7^	2.96	1.49
	2-methylbutanal	1.45	2.92 × 10^−2^	−1.12	1.31
	phenylacetaldehyde	1.20	9.06 × 10^−8^	++	+
esters	acetic acid ethyl ester	1.26	1.00 × 10^−6^	4.18	5.26
	lactic acid ethyl ester	1.37	2.05 × 10^−11^	3.48	2.69
isoflavones	glycitein	1.00	2.14 × 10^−3^	−4.37	−7.78
	genistein	1.33	8.49 × 10^−6^	−1.83	−4.69
	daidzein	1.16	3.15 × 10^−5^	−2.04	−2.77
other	isovaleronitrile	1.80	5.65 × 10^−5^	1.01	1.73
	uracil	1.15	2.45 × 10^−5^	1.33	1.40

VIP, variable importance in the projection; −, not detected; + and ++, newly generated; *p*-values were analyzed by Duncan’s test.

## Data Availability

Data are contained within the article and Supplementary Materials.

## Supplementary Materials

The following are available online at https://www.mdpi.com/article/10.3390/foods11010063/s1, Figure S1: Representative chromatograms of SSME metabolites analyzed by GC/MS (A: non-volatile compounds and B: volatile compounds), LC/MS (C), and HPLC (D), Figure S2: PLS-DA loading scatter plot, Table S1: Identification of major metabolites contributing to the separation of samples on the PLS-DA score plots by GC/MS analysis, Table S2: Identification of major metabolites contributing to the separation of samples on the PLS-DA score plots by UPLC-Q-TOF MS and HPLC, Table S3: Pearson correlation coefficient between microorganism and metabolites, Table S4: Pearson correlation coefficient between sensory qualities and metabolites.
