# Supplementary material for: Effects of Salt Treatment Time on the Metabolites, Microbial Composition, and Quality Characteristics of the Soy Sauce Moromi Extract
Source: Foods. 2021 Dec 28;11(1):63. doi: 10.3390/foods11010063 (PMC8750577; doi:10.3390/foods11010063)
Supplement: Supplementary file 1 [file foods-11-00063-s001.zip › foods-1492815-supplementary.pdf]

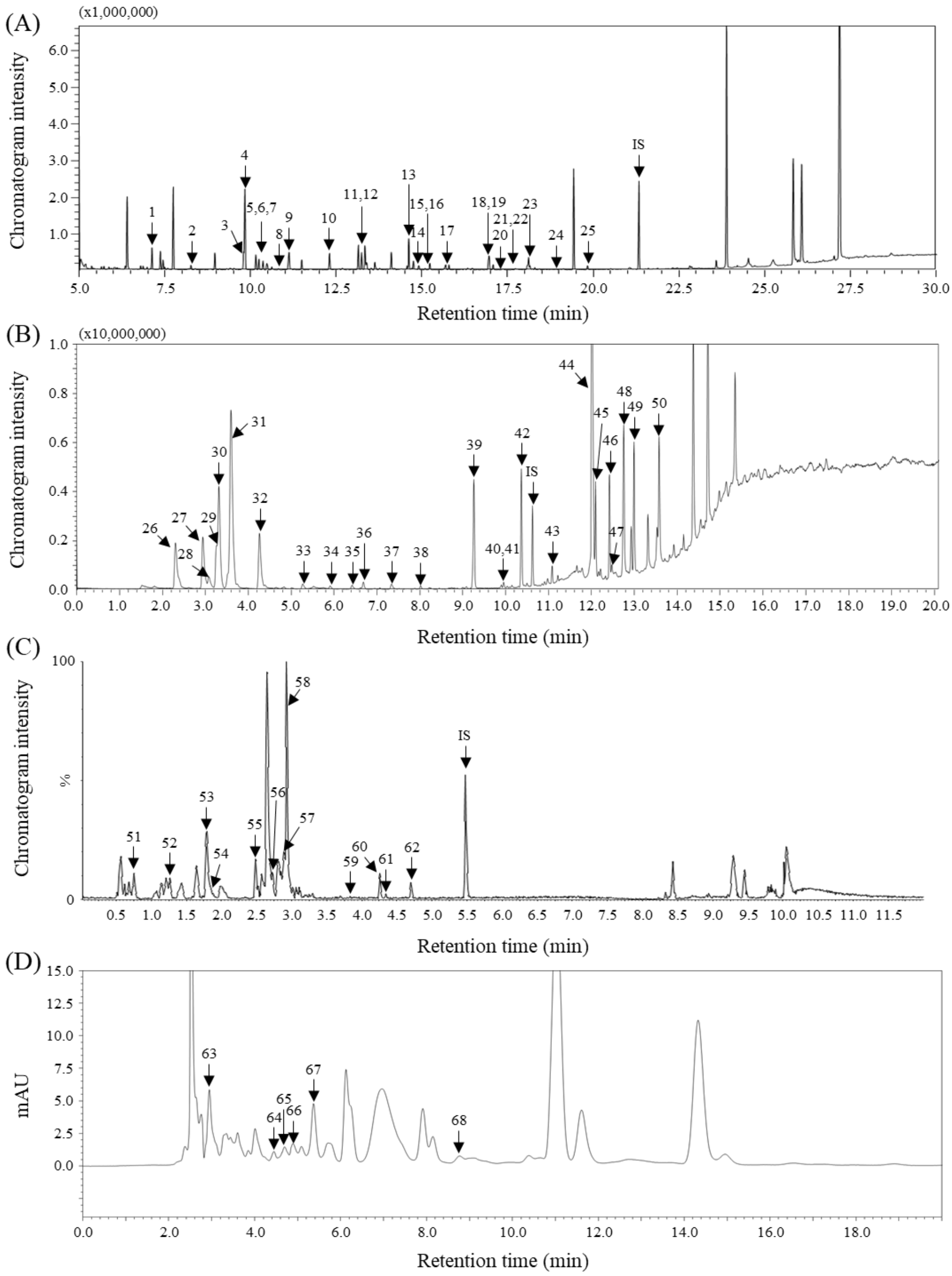

Figure S1. Representative chromatograms of citrus flesh metabolites analyzed by GC/MS (A: non-volatile compounds and B: volatile compounds), LC/MS (C), and HPLC (D). 1, alanine; 2, 2-aminobutanoic acid; 3, phosphoric acid; 4, glycerol; 5, isoleucine; 6, proline; 7, glycine; 8, uracil; 9, serine; 10; acetyl acetophenone; 11, meso-erythritol; 12, aspartic acid; 13, glutamic acid; 14, arabinose; 15, lyxose; 16, asparagine; 17, lysine; 18, arabitol; 19, citric acid; 20, tyropyranose; 21, mannose; 22, altronic acid; 23, mannitol; 24, galactonic acid; 25, myo-inositol; 26, acetone; 27, acetic acid ethyl ester; 28, 2-butanone; 29, 2-tehylbutanal; 30, 3-methylbutanal; 31, ethanol; 32, 2,3-butandione; 33, 2-butanol; 34, 2,3-pentanedione; 35, hexanal; 36, isobutanol; 37, isovaleronitrile; 38, 1-penten-3-ol; 39, isophenyl alcohol; 40, isopropenylethyl alcohol; 41, 1-pentanol; 42, acetoin; 43, lactic acid ethyl ester; 44, acetic acid; 45, 1-octen-3-ol; 46, 2-ethyl hexanol; 47, 1,2-butanediol; 48, 2,3-butanediol; 49, 1,3-butanediol; 50, isovaleric acid; 51, pro-arg; 52, glu-val; 53, phenylacetaldehyde; 54, cyclo(his-pro); 55, leu-glu; 56, tyr-pro; 57, trp-asp; 58, tryptophan; 59, glutamic acid 5-benzyl ester; 60, daidzein; 61, glycitein; 62, genistein; 63, oxalic acid; 64, malic acid; 65, ascorbic acid; 66, malonic acid; 67, lactic acid;68, succinic acid. IS: internal standard.

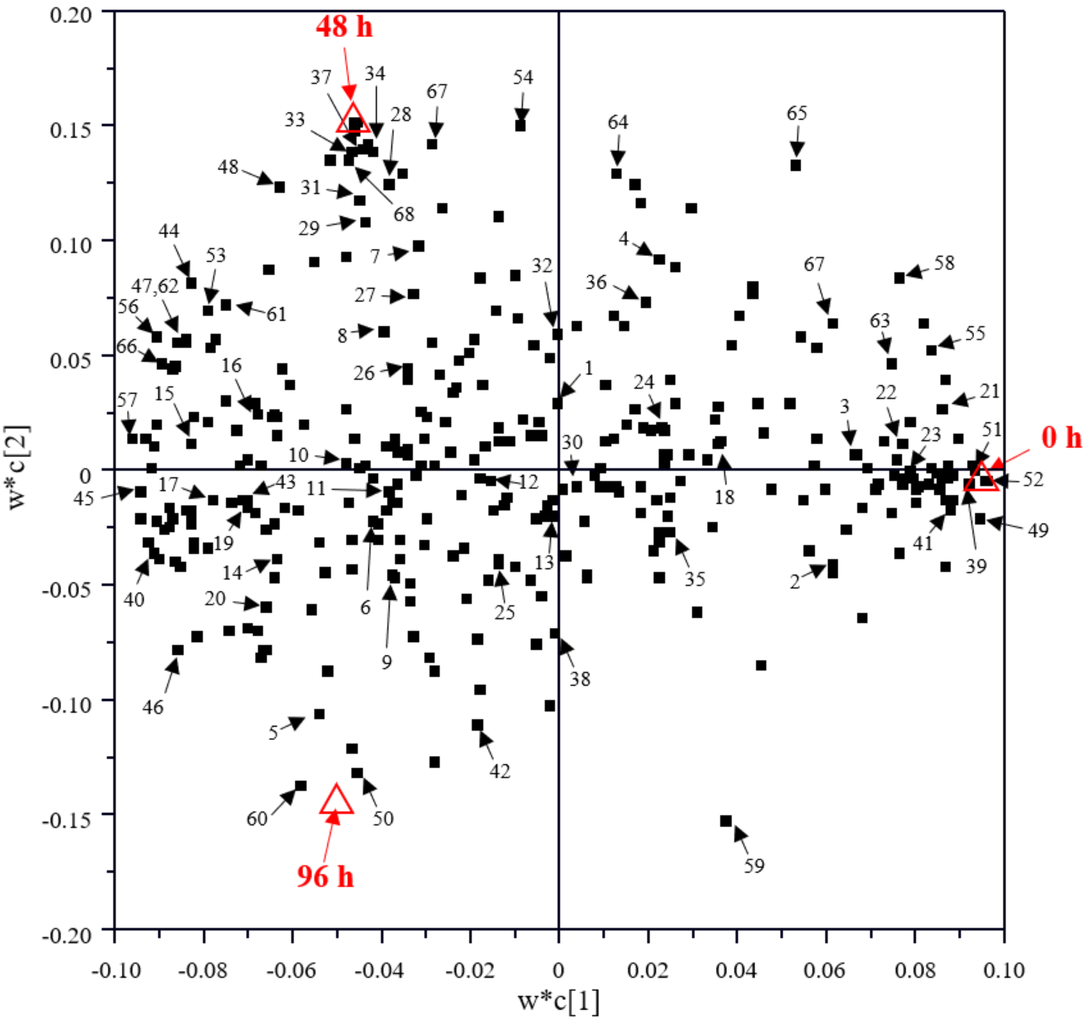

Figure S2. PLS-DA loading scatter plot. 1, alanine; 2, 2-aminobutanoic acid; 3, phosphoric acid; 4, glycerol; 5, isoleucine; 6, proline; 7, glycine; 8, uracil; 9, serine; 10; acetyl acetophenone; 11, meso-erythritol; 12, aspartic acid; 13, glutamic acid; 14, arabinose; 15, lyxose; 16, asparagine; 17, lysine; 18, arabitol; 19, citric acid; 20, tyropyranose; 21, mannose; 22, altronic acid; 23, mannitol; 24, galactonic acid; 25, myo-inositol; 26, acetone; 27, acetic acid ethyl ester; 28, 2-butanone; 29, 2-tehylbutanal; 30, 3-methylbutanal; 31, ethanol; 32, 2,3-butandione; 33, 2-butanol; 34, 2,3-pentanedione; 35, hexanal; 36, isobutanol; 37, isovaleronitrile; 38, 1-penten-3-ol; 39, isophenyl alcohol; 40, isopropenylethyl alcohol; 41, 1-pentanol; 42, acetoin; 43, lactic acid ethyl ester; 44, acetic acid; 45, 1-octen-3-ol; 46, 2-ethyl hexanol; 47, 1,2-butanediol; 48, 2,3-butanediol; 49, 1,3-butanediol; 50, isovaleric acid; 51, pro-arg; 52, glu-val; 53, phenylacetaldehyde; 54, cyclo(his-pro); 55, leu-glu; 56, tyr-pro; 57, trp-asp; 58, tryptophan; 59, glutamic acid 5-benzyl ester; 60, daidzein; 61, glycitein; 62, genistein; 63, oxalic acid; 64, malic acid; 65, ascorbic acid; 66, malonic acid; 67, lactic acid;68, succinic acid.

Table S1. Identification of major metabolites contributing to the separation of samples on the PLS-DA score plots by GC/MS analysis.

	RT	Compounds	RI
non-volatile conpounds	7.12	alanine	1073
	8.26	2-aminobutyric acid	1150
	9.80	phosphoric acid	1265
	9.84	glycerol	1268
	10.15	isoleucine	1288
	10.24	proline	1293
	10.36	glycine	1301
	10.81	uracil	1331
	11.12	serin	1351
	12.30	acetyl acetophenone	1434
	13.24	meso-erythritol	1506
	13.34	aspartic acid	1513
	14.61	glutamic acid	1613
	14.90	arabinose	1636
	15.09	lyxose	1652
	15.23	asparagine	1663
	15.67	lysine	1699
	16.85	ornithine	1801
	16.94	citric acid	1810
	17.36	talopyranose	1848
	17.72	mannose	1880
	17.79	altronic acid	1886
	18.15	mannitol	1920
	18.90	galactonic acid	1992
	19.82	myo-inositol	2083
volatile compounds	2.31	acetone	821
	2.94	acetic acid ethyl ester	885
	3.09	2-butanone	917
	3.26	2-methylbutanal	906
	3.31	3-methylbutanal	925
	3.60	ethanol	926
	4.27	2,3-butanedione	977
	5.28	2-butanol	1030
	5.90	2,3-pentanedione	1060
	6.42	hexanal	1079
	6.68	isobutanol	1150
	7.38	5-methyl-3-hexen-2-one	1152
	8.22	1-penten-3-ol	1157
	9.26	isopentyl alcohol	1211
	9.90	isopropenylethyl alcohol	1250
	9.95	1-pentanol	1261
	10.37	acetoin	1285
	11.09	lactic acid ethyl ester	1340
	12.04	acetic acid	1415
	12.10	1-octen-3-ol	1456
	12.42	2-ethyl hexanol	1492
	12.51	1,2-butanediol	1563
	12.76	2,3-butanediol	1583
	13.00	1,3-butanediol	1578
	13.58	isovaleric acid	1680

RT, retention time; RI: retention index.

Table S2. Identification of major metabolites contributing to the separation of samples on the PLS-DA score plots by UPLC-Q-TOF MS and HPLC.

	**RT**	**Compounds**	**Exact Mass (m/z)**	**MS Fragments**
UPLC-Q-TOF MS	0.75	pro-arg	272.1641	70, 116, 130
	1.26	glu-val	247.1264	72
	1.77	phenylacetaldehyde	121.0621	77, 103, 91
	1.80	cyclo(his-pro)	235.1153	110
	2.48	leu-glu	261.1424	86
	2.71	tyr-pro	279.1319	116, 136, 181, 119
	2.80	trp-asp	320.1176	159, 303, 144, 170
	2.92	tryptophan	188.0681	188, 146, 118
	3.83	glutamic acid 5-benzyl ester	238.1020	
	4.25	daidzein	255.0624	237, 137
	4.34	glycitein	285.0717	270
	4.70	genistein	271.0562	153, 253
HPLC	3.13	oxalic acid		
	4.40	malic acid		
	4.80	ascorbic acid		
	4.87	malonic acid		
	5.36	lactic acid		
	8.78	succinic acid		

RT, retention time.

Table S3. Pearson correlation coefficient between microorganism and metabolites.

		Enterococcaceae	*Lactobacillaceae*	*Staphylococcaceae*	*Bacillaceae*	*Enterobacteriaceae*
sugar	meso-erythritol	0.85	0.94	-0.91	-0.95	-0.95
	myo-inositol	0.67	0.81	-0.76	-0.83	-0.84
	lyxose	0.56	0.70	-0.64	-0.71	-0.72
	arabinose	-0.68	-0.71	0.70	0.71	0.72
	mannose	-0.68	-0.68	0.68	0.67	0.67
	talopyranose	-0.71	-0.74	0.73	0.74	0.74
	altronic acid	-0.78	-0.88	0.84	0.89	0.89
	mannitol	-0.93	-0.91	0.92	0.90	0.90
	galactonic acid	-0.95	-0.99	0.98	0.99	0.99
acidic compounds	acetic acid	0.96	0.99	-0.99	-0.99	-0.99
	ascorbic acid	0.96	0.94	-0.96	-0.93	-0.93
	lactic acid	0.71	0.54	-0.62	-0.52	-0.50
	malonic acid	0.54	0.36	-0.44	-0.33	-0.31
	citric acid	0.50	0.65	-0.59	-0.67	-0.68
	phosphoric acid	0.33	0.18	-0.25	-0.16	-0.15
	oxalic acid	0.11	-0.08	0.00	0.10	0.12
	malic acid	-0.28	-0.49	0.41	0.52	0.54
	succinic acid	-0.49	-0.61	0.56	0.62	0.63
	isovaleric acid	-0.65	-0.76	0.72	0.77	0.77
amino acids and peptides	leu-glu	0.93	0.89	-0.91	-0.88	-0.87
	aspartic acid	0.88	0.96	-0.94	-0.97	-0.97
	trp-asp	0.86	0.82	-0.84	-0.81	-0.80
	glycine	0.85	0.93	-0.90	-0.93	-0.94
	alanine	0.83	0.90	-0.88	-0.91	-0.91
	isoleucine	0.82	0.91	-0.88	-0.91	-0.92
	glutamic acid 5-benzyl ester	0.82	0.83	-0.83	-0.82	-0.82
	ornithine	0.81	0.89	-0.86	-0.90	-0.90
	tryptophan	0.79	0.86	-0.84	-0.86	-0.87
	glutamic acid	0.78	0.88	-0.84	-0.89	-0.89
	2-aminobutyric acid	0.77	0.85	-0.82	-0.85	-0.86
	proline	0.70	0.76	-0.74	-0.76	-0.76
	tyr-pro	0.65	0.66	-0.66	-0.66	-0.66
	serin	0.60	0.74	-0.68	-0.75	-0.76
	glu-val	0.49	0.36	-0.42	-0.35	-0.33
	lysine	0.23	0.43	-0.35	-0.46	-0.47
	asparagine	0.06	-0.13	0.05	0.15	0.17
	pro-arg	-0.29	-0.42	0.36	0.43	0.44
	cyclo(his-pro)	-0.85	-0.89	0.88	0.89	0.89
alcohols	ethanol	0.96	0.88	-0.92	-0.87	-0.86
	1,3-butanediol	0.94	0.91	-0.93	-0.90	-0.89
	isopropenylethyl alcohol	0.91	0.85	-0.88	-0.84	-0.83
	2,3-butanediol	0.87	0.81	-0.84	-0.79	-0.78
	glycerol	0.76	0.86	-0.82	-0.87	-0.88
	2-butanol	0.69	0.85	-0.79	-0.87	-0.88
	1-pentanol	0.36	0.15	-0.24	-0.13	-0.11
	1,2-butanediol	0.32	0.54	-0.45	-0.57	-0.59
	1-octen-3-ol	-0.60	-0.76	0.70	0.78	0.79
	2-ethyl hexanol	-0.65	-0.45	0.54	0.42	0.40
	1-penten-3-ol	-0.91	-0.96	0.95	0.96	0.96
	isopentyl alcohol	-0.95	-0.99	0.98	1.00	1.00
	isobutanol	-0.96	-0.98	0.98	0.98	0.98
ketones	2,3-pentanedione	0.92	0.89	-0.91	-0.88	-0.87
	2,3-butanedione	0.91	0.97	-0.95	-0.97	-0.97
	acetyl acetophenone	0.51	0.66	-0.60	-0.67	-0.69
	acetoin	-0.73	-0.84	0.80	0.86	0.86
	2-butanone	-0.89	-0.92	0.91	0.91	0.91
	acetone	-0.92	-0.96	0.95	0.96	0.96
aldehydes	phenylacetaldehyde	0.94	0.97	-0.96	-0.97	-0.96
	hexanal	0.84	0.70	-0.76	-0.68	-0.66
	3-methylbutanal	0.78	0.84	-0.82	-0.85	-0.85
	2-methylbutanal	-0.02	0.15	-0.08	-0.17	-0.18
esters	lactic acid ethyl ester	0.99	0.96	-0.98	-0.95	-0.94
	acetic acid ethyl ester	0.82	0.92	-0.89	-0.93	-0.94
isoflavones	daidzein	-0.69	-0.82	0.77	0.83	0.84
	glycitein	-0.74	-0.79	0.77	0.79	0.80
	genistein	-0.80	-0.88	0.85	0.89	0.89
other	uracil	0.81	0.89	-0.87	-0.90	-0.90
	isovaleronitrile	0.21	0.42	-0.33	-0.44	-0.46

Table S4. Pearson correlation coefficient between sensory qualities and metabolites.

		Umami	Sourness	Salty	Acetic Acid Odor	Bitterness	Ethanol Odor	Soy Sauce Odor	Sweetness
sugar	lyxose	0.95	0.70	0.64	0.82	0.80	0.46	0.45	0.59
	myo-inositol	0.93	0.76	0.64	0.80	0.71	0.33	0.47	0.46
	meso-erythritol	0.87	0.86	0.79	0.79	0.58	0.32	0.30	0.23
	mannose	-0.24	-0.46	-0.45	-0.26	0.08	0.17	0.10	0.33
	arabinose	-0.29	-0.43	-0.40	-0.23	0.12	0.27	0.07	0.31
	talopyranose	-0.29	-0.45	-0.43	-0.23	0.07	0.16	0.11	0.34
	mannitol	-0.42	-0.68	-0.71	-0.29	0.04	-0.05	0.28	0.45
	altronic acid	-0.55	-0.56	-0.53	-0.37	-0.12	0.14	0.05	0.04
	galactonic acid	-0.69	-0.83	-0.80	-0.60	-0.31	-0.19	-0.02	0.09
acidic conpounds	citric acid	0.91	0.71	0.57	0.83	0.82	0.42	0.59	0.62
	acetic acid	0.76	0.90	0.88	0.69	0.43	0.34	0.06	-0.02
	ascorbic acid	0.70	0.94	0.93	0.75	0.44	0.47	0.06	-0.09
	lactic acid	0.28	0.73	0.83	0.50	0.25	0.64	-0.21	-0.33
	malonic acid	0.20	0.61	0.73	0.43	0.28	0.75	-0.23	-0.29
	phosphoric acid	0.16	0.47	0.54	0.47	0.44	0.66	-0.03	0.02
	oxalic acid	-0.15	0.27	0.30	0.21	0.04	0.54	-0.03	-0.35
	succinic acid	-0.30	-0.20	-0.18	0.01	0.13	0.47	0.17	0.06
	isovaleric acid	-0.43	-0.45	-0.36	-0.30	-0.01	0.31	-0.09	0.08
	malic acid	-0.57	-0.19	-0.09	-0.23	-0.23	0.31	-0.27	-0.41
amino acids and peptides	serin	0.95	0.78	0.66	0.84	0.78	0.39	0.53	0.57
	glutamic acid	0.93	0.87	0.78	0.86	0.69	0.42	0.38	0.36
	tryptophan	0.91	0.93	0.86	0.88	0.75	0.56	0.37	0.34
	glycine	0.91	0.91	0.84	0.82	0.63	0.41	0.29	0.26
	2-aminobutyric acid	0.91	0.91	0.84	0.82	0.67	0.45	0.32	0.37
	ornithine	0.88	0.88	0.80	0.86	0.67	0.41	0.36	0.30
	proline	0.88	0.87	0.78	0.92	0.79	0.56	0.41	0.44
	isoleucine	0.87	0.88	0.80	0.82	0.64	0.37	0.32	0.29
	alanine	0.86	0.89	0.82	0.83	0.66	0.41	0.29	0.29
	aspartic acid	0.84	0.88	0.81	0.75	0.53	0.31	0.22	0.16
	glutamic acid 5-benzyl ester	0.79	0.88	0.85	0.85	0.62	0.50	0.21	0.21
	tyr-pro	0.79	0.86	0.80	0.92	0.80	0.67	0.38	0.41
	lysine	0.75	0.37	0.24	0.61	0.68	0.08	0.56	0.79
	leu-glu	0.69	0.94	0.97	0.73	0.51	0.54	-0.01	0.01
	trp-asp	0.69	0.88	0.93	0.78	0.57	0.58	0.01	0.09
	glu-val	0.38	0.60	0.74	0.59	0.57	0.80	-0.06	0.09
	asparagine	-0.09	0.25	0.35	0.28	0.24	0.66	-0.04	-0.13
	pro-arg	-0.12	0.00	0.10	0.04	0.22	0.55	-0.05	0.17
	cyclo(his-pro)	-0.52	-0.67	-0.63	-0.47	-0.16	-0.02	0.00	0.18
alcohols	glycerol	0.91	0.87	0.76	0.87	0.70	0.39	0.44	0.39
	2-butanol	0.87	0.70	0.59	0.67	0.51	0.13	0.36	0.34
	1,2-butanediol	0.78	0.40	0.22	0.55	0.50	-0.06	0.57	0.58
	1,3-butanediol	0.71	0.96	0.95	0.75	0.51	0.60	0.06	-0.10
	isopropenylethyl alcohol	0.67	0.94	0.94	0.73	0.49	0.62	0.01	-0.10
	2,3-butanediol	0.66	0.95	0.95	0.74	0.51	0.64	0.05	-0.06
	ethanol	0.58	0.90	0.95	0.63	0.37	0.52	-0.14	-0.20
	2-ethyl hexanol	0.14	-0.41	-0.57	0.02	0.29	-0.23	0.62	0.80
	1-pentanol	-0.01	0.46	0.56	0.30	0.22	0.72	-0.20	-0.33
	1-octen-3-ol	-0.59	-0.45	-0.35	-0.34	-0.14	0.25	-0.19	-0.09
	1-penten-3-ol	-0.61	-0.73	-0.71	-0.45	-0.15	-0.08	0.08	0.21
	isobutanol	-0.61	-0.80	-0.80	-0.52	-0.22	-0.17	0.09	0.20
	isopentyl alcohol	-0.70	-0.83	-0.81	-0.59	-0.31	-0.20	-0.01	0.09
ketones	acetyl acetophenone	0.92	0.71	0.55	0.86	0.80	0.37	0.66	0.60
	2,3-butanedione	0.84	0.90	0.84	0.77	0.53	0.35	0.21	0.11
	2,3-pentanedione	0.69	0.93	0.95	0.78	0.53	0.56	0.07	-0.01
	2-butanone	-0.49	-0.67	-0.68	-0.34	-0.03	-0.05	0.16	0.36
	acetoin	-0.59	-0.55	-0.47	-0.42	-0.16	0.15	-0.13	0.02
	acetone	-0.61	-0.75	-0.73	-0.46	-0.17	-0.14	0.04	0.24
aldehydes	3-methylbutanal	0.88	0.91	0.81	0.91	0.73	0.54	0.47	0.28
	phenylacetaldehyde	0.78	0.92	0.89	0.77	0.52	0.40	0.14	0.07
	2-methylbutanal	0.65	0.21	0.13	0.61	0.69	0.14	0.63	0.90
	hexanal	0.38	0.80	0.91	0.52	0.31	0.64	-0.27	-0.31
esters	acetic acid ethyl ester	0.88	0.84	0.77	0.80	0.58	0.29	0.30	0.28
	lactic acid ethyl ester	0.64	0.92	0.95	0.62	0.34	0.44	-0.10	-0.20
isoflavones	glycitein	-0.33	-0.46	-0.42	-0.23	0.06	0.25	0.10	0.26
	genistein	-0.53	-0.57	-0.56	-0.33	-0.07	0.09	0.06	0.17
	daidzein	-0.59	-0.51	-0.43	-0.38	-0.16	0.18	-0.12	-0.03
other	uracil	0.92	0.92	0.86	0.85	0.70	0.50	0.32	0.32
	isovaleronitrile	0.84	0.40	0.26	0.64	0.69	0.17	0.63	0.76
